# Karyotype analysis of *Panax ginseng* C.A.Meyer, 1843 (Araliaceae) based on rDNA loci and DAPI band distribution

**DOI:** 10.3897/CompCytogen.v6i4.3740

**Published:** 2012-12-15

**Authors:** Nomar Espinosa Waminal, Hye Mi Park, Kwang Bok Ryu, Joo Hyung Kim, Tae-Jin Yang, Hyun Hee Kim

**Affiliations:** 1 Department of Plant Science, Plant Genomics and Breeding Institute, and Research Institute for Agriculture and Life Sciences, College of Agriculture and Life Sciences, Seoul National University, Seoul, 151–921, Korea; 2 Plant Biotechnology Institute, Department of Life Science, Sahmyook University, Seoul, 139–742, Korea; 3 Department of Horticultural Science, Kyungpook National University, Daegu, 702–701, Korea

**Keywords:** *Panax ginseng*, FISH, 5SrDNA, 45S rDNA, DAPI band, Araliaceae

## Abstract

Ginseng has long been considered a valuable plant owing to its medicinal properties; however, genomic information based on chromosome characterization and physical mapping of cytogenetic markers has been very limited. Dual-color FISH karyotype and DAPI banding analyses of *Panax ginseng* C. A. Meyer, 1843 were conducted using 5S and 45S rDNA probes. The somatic chromosome complement was 2*n*=48 with lengths from 3.3 μm to 6.3 μm. The karyotype was composed of 12 metacentric, 9 submetacentric, and 3 subtelocentric pairs. The 5S rDNA probe localized to the intercalary region of the short arm of pair 11, while the 45S rDNA was located at the secondary constriction of the subtelocentric satellited chromosome 14. DAPI bands were clearly observed for most chromosomes, with various signal intensities and chromosomal distributions that consequently improved chromosome identification. As a result, all 24 chromosomes could be distinguished and numbers were assigned to each chromosome for the first time. The results presented here will be useful for the on-going ginseng genome sequencing and further molecular-cytogenetic studies and breeding programs of ginseng.

## Introduction

Ginseng (*Panax ginseng* C.A.Meyer, 1843) is highly valued owing to its medicinal properties (Zhang et al. 2011), and the ginsenosides found in the plant contribute greatly to its pharmacological value (Court 2000, Leung and Wong 2010, Yuan et al. 2010). Along with 15–17 other species, ginseng belongs to the genus *Panax* in the family Araliaceae (Ho and Leung 2002, Yi et al. 2004). This genus is only one of the approximately 120 genera of angiosperms with a disjunct distribution pattern between eastern North America and eastern Asia (Wen and Zimmer 1996). Most of the species of *Panax* are geographically distributed in eastern Asia, but two (*Panax trifolius* Linnaeus, 1753 and *Panax quinquefolius* Linnaeus, 1753) are isolated in eastern North America (Ho and Leung 2002). American ginseng (*Panax quinquefolius*) is morphologically similar to ginseng (Ngan et al. 1999), and both are regarded as polyploid (Court 2000); however, their origin (auto- vs. allopolyploidy) is not yet fully understood (Yi et al. 2004, Choi et al. 2009). Cytogenetic data have been employed in an attempt to explain the possible origins of their disjunct distribution (Yang 1981, Wen and Zimmer 1996, Yi et al. 2004), but these did not sufficiently resolve the question. Apparently, more research is needed to fully understand their phylogenetic relationship.

Information regarding the chromosome number of ginseng has been available since 1936 (Darlington and Wylie 1956, Yi et al. 2004). However, data reported by different researchers have been inconsistent. For example, Graham (1966) and Yang (1981) reported 2*n*=44, while Ko et al. (1993) and Choi et al. (2009) reported a complement of 2*n*=48. Regardless of whether or not the discrepancy in the reported chromosome number is caused by intraspecific variation (Blair 1975), it is essential to establish a detailed karyotype for ginseng.

The translocation of DNA blocks in some plants have been observed through cytogenetic investigations (e.g. Han et al. 2009, Huang et al. 2009, Topp et al. 2009), and helped us to understand the genomic relationships among several plants (Leflon et al. 2006, Snowdon 2007, Xiong and Pires 2011, Chester et al. 2012), making cytogenetics an essential tool to the overall understanding of a genome. Moreover, fluorescence *in situ* hybridization (FISH) is an excellent technique for use in plant cytogenetics (Sadder and Weber 2001, Capdeville et al. 2008, Vasconcelos et al. 2010) because it allows physical mapping of a particular DNA sequence along the chromosome complement. Examples include the repetitive sequences of ribosomal RNA genes (rDNA), centromeric and telomeric repeats (e.g. Kato et al. 2004, Lim et al. 2005), and single-copy genes (e.g. Fransz et al. 1996, Kharb et al. 2001). Owing to their sequence conservation among eukaryotic genomes despite the repeating unit copy number, loci number, and distribution pattern variations, the multiple tandem repeats of the 5S and 45S rDNA are the most widely used probes in molecular cytogenetic analyses (e.g. Chen et al. 1999, Hwang et al. 2009, Waminal et al. 2011). Indeed, these cytogenetic markers are invaluable in cytogenetic studies such as karyotyping, investigations of chromosomal organizational changes, and physical mapping of DNA sequences (Huang et al. 2009, Park et al. 2012).

Probes labeled with different fluorophores for simultaneous detection have been widely employed in rDNA loci distribution analyses and dual-color FISH karyotyping (e.g. Ali et al. 2005, Lan and Albert 2011, Xiang-Hui 2011, Waminal and Kim 2012). Choi et al. (2009) recently reported the number of rDNA loci in ginseng using dual-color FISH; however, no detailed karyotype or chromosome characterization was presented. To date, molecular cytogenetic information pertaining to ginseng, despite recent development of molecular markers (Choi et al. 2011, Kim et al. 2012a, 2012b), has been very limited causing the slow progress of genomic studies.

Here, we used dual-color FISH to analyze the distribution of rDNA loci in *Panax ginseng*. In addition, we used the DAPI banding pattern to pair homologous chromosomes. Collectively, this made numbering of the chromosome of *Panax ginseng* possible for the first time. These data will be useful for future cytogenetic analyses and should enable a better understanding of the genomic history of ginseng, and can be used for subsequent distribution analyses of repeat sequences, retrotransposons, and chromosome-specific cytogenetic markers. Consequently, the results presented here will make a significant contribution to studies related to the on-going ginseng genome sequencing and the overall understanding of the *Panax ginseng* genome.

## Material and methods

### Root sample preparation

Stratified seeds of three ginseng cultivars ‘Sunun’, ‘Chunpoong’, ‘Gopoong’, and a local landrace ‘Hwangsook’ were provided by the Korea Ginseng Corporation (KGC) Natural Resources Research Institute (Daejeon, Korea). Stratified seeds were allowed to germinate in petri dishes with wet filter papers at 10–15°C. The root meristems were then excised (about 2 cm from the root tips), pretreated with 0.002M 8-hydroxyquinoline for 5 hours at 18°C, fixed in 90% acetic acid for 15 min at room temperature (RT, ~24°C), and then stored in 70% ethanol until use.

### Chromosome spread preparation

Somatic chromosome spreads were obtained using a modified version of the technique described by Kato et al. (2004). After thorough washing with distilled water, the meristematic regions of the fixed root tips (~2 mm) were excised and digested in a pectolytic enzyme mix [2% cellulase (MB Cell, Korea), 1.5% macerozyme (Maxim Bio, USA) and 1% pectolyase (Sigma, Japan) in 150 mM Citrate Buffer, pH 4.5] for 75 min at 37°C. The digested meristems were then pipetted into a petri dish with chilled distilled water and incubated on ice for 15 min to wash out the enzymes. Using a stereomicroscope, the root epidermis was removed, and the protoplasts were gently pipetted into a 1.5 ml tube containing 40 μl chilled Carnoy’s fixative. The protoplasts were then suspended by gently vortexing the tube for 30 sec at room temperature, after which the sample was centrifuged at 4,000 ×*g* for 3 min and the pellet was resuspended in acetic acid-ethanol (9:1) solution. Finally, the protoplast suspension was pipette-mounted onto ethanol cleaned glass slides, which were placed in a humid chamber to facilitate spreading of the chromosomes and allowed to dry.

### Probe labeling

A 9-kb fragment of 45S rDNA (18S-5.8S-25S) (Gerlach and Bedbrook 1979) was labeled with biotin-16-dUTP (Roche, Germany) by nick translation. The 5S rDNA was obtained according to the procedure described by Hwang et al. (2009) and then labeled with digoxigenin-11-dUTP (Roche, Germany) by nick translation. Labeled DNA fragments within the range of 200–500 bp were used as probes.

### Fluorescence *in situ* hybridization

*Slide pretreatment*. To remove contaminating RNA, the slides were treated with RNase A buffer (RNase A final conc. 100 μg ml^-1^ in 2× SSC) for 1 hr at 37°C. The slides were then incubated in 0.01 M HCl for two minutes, followed by subsequent treatment in pepsin buffer [stock: 10% (w/v) pepsin in dH_2_O, working: 1:100 dilution in 0.01 M HCl] for 10 min at 37°C to lyse endogenous proteins that could cause background signals. Next, the chromosomes were fixed by treating the slides with 4% paraformaldehyde in 2× SSC. Finally, the slides were dehydrated in ethanol series (70%, 90%, 100%, 3 min each) and air-dried. The slides were washed in 2× SSC for 5 min (3×) between each step. All incubation steps at 37°C were conducted in a humidified chamber.

*Probe hybridization*.The hybridization mixture contained 50% formamide, 10% dextran sulfate, 2× SSC, 5 ng μl^-1^ salmon sperm DNA and 500 ng μl^-1^ of each probe DNA adjusted with DNase- and RNase-free water (Sigma, USA, #W4502) to a total volume of 40 μl/slide. The mixture was denatured at 90°C for 10 min and immediately kept on ice for at least 5 min prior to mounting on slides. After covering with a glass cover slip, the chromosomes were denatured at 80°C for 3–5 min on a hot plate. The slides were then immediately transferred into a humid chamber preset at 37°C and incubated overnight (~16 hr). The following day, the slides were washed in 2× SSC (15 min at RT), 0.1× SSC (35 min at 42°C), and finally 2×SSC (30 min at RT).

*Signal detection*. The slides were treated with TNB [0.1 M Tris-HCl, 0.15 M NaCl, 1% (w/v) blocking reagent] at RT for 5 min, after which they were subjected to antibody detection. Briefly, biotinylated 45S rDNA probe was detected with streptavidin-Cy3 conjugate (Zymed, USA), while digoxigenin-labeled 5S rDNA probe was detected using anti-digoxigenin-FITC conjugate (Sigma, USA). Both antibodies were diluted in TNB to a ratio of 1:100, and the slides were then incubated at 37°C for one hour. Excess reagents were subsequently washed off in TNT [0.1 M Tris-HCl, 0.15 M NaCl, 0.2% (v/v) Tween-20] at 37°C for 5 min (3×), after which they were subjected to dehydration in ethanol series (70%, 90%, 100%, 3 min each) and air-dried. Chromosomes were then counterstained with a premixed DAPI solution [1 μg ml^-1^ DAPI in Vectashield (Vector Laboratories, USA)].

## Karyotyping

*Image capture and measurement*. Well-spread chromosomes with well-preserved chromosome morphology were observed and captured using an Olympus BX51 fluorescence microscope equipped with a CCD camera (CoolSNAP™ cf) and filters for DAPI, FITC, and Cy3. The captured FISH images were analyzed, after which each homologue was measured 3–7 times using Genus™ version 3.1 (Applied Imaging, USA) to obtain the mean values. Raw images for each probe were saved separately and a pseudo-colored image of the merged signals was obtained for each chromosome spread. The sharpness value in Genus™ was set to 7 to enhance the details and texture of the chromosomes. Final images were edited using Adobe Photoshop CS3.

*Chromosome numbering and pairing*. Chromosome number assignment was based on the decreasing order of chromosome lengths, while homologous chromosome pairing was achieved according to the centromeric position (Levan et al. 1964), DAPI band and rDNA loci distribution. Chromosomes were grouped according to the number of DAPI bands in each arm. As demonstrated by Costa Silva et al. (2011), the estimated DNA content in each chromosome was calculated by distributing the 1C DNA content of *Panax ginseng* (3.12×10^3^ Mb, Hong et al. 2004) relative to the length of each chromosome.

## Results

### Chromosome complement composition and rDNA localization

**Figure 1.**
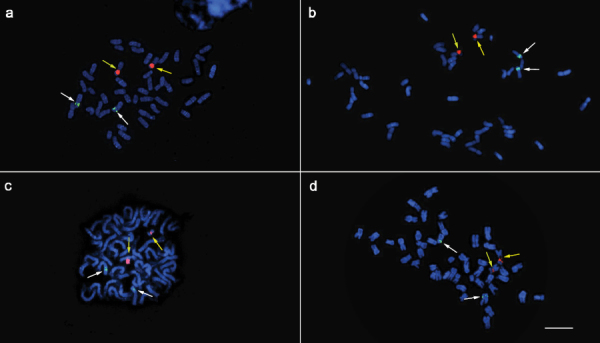
Chromosome complement of three *Panax ginseng* cultivars, ‘Sunun’ (**a**), ‘Gopoong’ (**b**), ‘Chunpoong’ (**c**), and one local landrace, ‘Hwangsook’ (**d**) showing 2*n*=48. One pair of 45S rDNA (red signals, yellow arrows) and one pair of 5S rDNA (green signals, white arrows) was observed among the four samples. Bar = 10 μm.

The three cultivars and one landrace of *Panax ginseng* evaluated in this study were all confirmed to have a chromosome complement of 2*n*=48 (Fig. 1). With reference to the centromere position (i.e. arm ratio), the complement comprised 12 metacentric (1–7, 11–13, 15, and 18), 9 submetacentric (8–10, 16–17, 19, and 22–24), and 3 subtelocentric (14 and 20–21) homologous pairs with a karyotype formula of 24m+18sm+6st. The chromosome lengths ranged from 3.27 to 6.30 μm (Table 1).

**Table 1.** Chromosome analyses of *Panax ginseng* based on chromosome length and centromeric position.

Chr. no.	Chr. length (μm)			Arm ratio (q/p)	Type
	Short arm (p)	Long arm (q)	Total		
1	3.16 ± 0.12	3.17 ± 0.11	6.30 ± 0.22	1.002	m
2	2.64 ± 0.08	3.27 ± 0.12	6.05 ± 0.06	1.237	m
3	2.52 ± 0.23	3.61 ± 0.11	5.88 ± 0.18	1.434	m
4	2.54 ± 0.20	3.27 ± 0.22	5.64 ± 0.07	1.289	m
5	2.23 ± 0.27	3.35 ± 0.12	5.41 ± 0.17	1.506	m
6	2.05 ± 0.11	3.30 ± 0.06	5.31 ± 0.23	1.609	m
7	2.09 ± 0.07	3.35 ± 0.22	5.30 ± 0.14	1.605	m
8	1.54 ± 0.32	3.66 ± 0.13	5.23 ± 0.40	2.378	sm
9	1.52 ± 0.19	3.82 ± 0.13	5.08 ± 0.21	2.515	sm
10	1.77 ± 0.04	3.49 ± 0.06	5.04 ± 0.28	1.965	sm
11†	2.13 ± 0.12	2.91 ± 0.13	4.94 ± 0.12	1.363	m
12	1.96 ± 0.07	3.03 ± 0.12	4.83 ± 0.28	1.547	m
13	2.04 ± 0.05	3.05 ± 0.04	4.82 ± 0.07	1.492	m
14‡	1.99§ ± 0.21	3.21 ± 0.14	4.80 ± 0.31	1.612|	st
15	2.26 ± 0.17	2.58 ± 0.28	4.73 ± 0.49	1.143	m
16	1.55 ± 0.09	3.33 ± 0.10	4.72 ± 0.08	2.157	sm
17	1.59 ± 0.15	3.05 ± 0.07	4.50 ± 0.11	1.919	sm
18	2.09 ± 0.25	2.54 ± 0.19	4.50 ± 0.06	1.214	m
19	1.39 ± 0.12	2.78 ± 0.17	4.11 ± 0.21	1.998	sm
20	1.05 ± 0.04	3.24 ± 0.07	4.09 ± 0.06	3.067	st
21	0.90 ± 0.05	3.02 ± 0.21	3.80 ± 0.13	3.355	st
22	1.32 ± 0.06	2.32 ± 0.10	3.56 ± 0.09	1.761	sm
23	1.25 ± 0.11	2.30 ± 0.22	3.38 ± 0.09	1.836	sm
24	1.13 ± 0.25	2.08 ± 0.24	3.27 ± 0.10	1.840	sm

†5S rDNA, ‡45S rDNA, ^§^satellite length, ^|^value obtained using satellite instead of short arm, m: metacentric, sm: submetacentric, st: subtelocentric (Levan et al. 1964)

**Figure 2.**
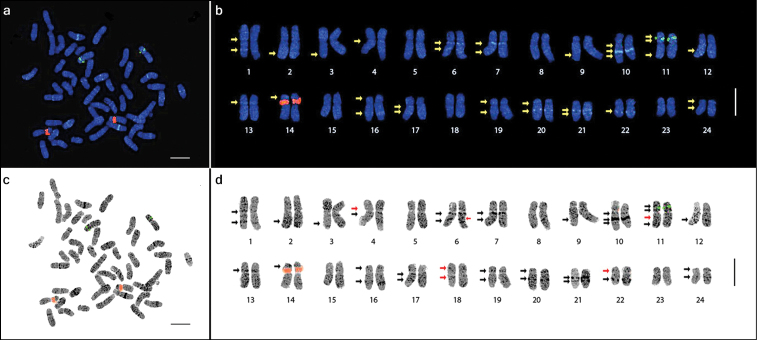
Metaphase spread of *Panax ginseng* 2*n*=48 chromosomes (**a** and **c**) and the karyotype idiogram showing 24 homologous pairs (enlarged; **b** and **d**) arranged in decreasing lengths. The 5S and 45S rDNA loci are shown as green and red signals, respectively. DAPI bands (arrows) were detected in various intensities and inverse images (**c** and **d**) were obtained to emphasize these DAPI bands. Note the heterochromatic dots (dark dots in **d**). The red arrows in **d** indicate the six bands observed after inversing the image. Bar=5 μm.

**Figure 3.**
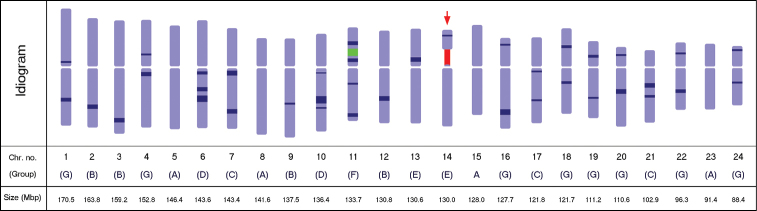
Diagrammatic idiogram of the *Panax ginseng* karyotype showing the 5S (green) and the 45S (red) rDNA loci, and the 38 observed DAPI bands (dark blue), 12 on the short arm and 26 on the long arm. The satellited chromosome is indicated by the red arrow. DAPI band depths indicate relative intensities. Chromosomes were grouped according to the DAPI band pattern on each arm. The estimated relative size of each chromosome is presented in mega base-pairs.

Only one pair of satellited chromosomes (pair 14) was observed, and the only locus of 45S rDNA in the genome was localized at the secondary constriction of this subtelocentric chromosome (Figs 2, 3 and Table 2). Moreover, one locus of 5S rDNA signal was detected at the intercalary region of the short arm of chromosome 11. This locus was flanked by two DAPI bands. There was no variation in the number of rDNA loci among the three cultivars and one landrace of *Panax ginseng* investigated in this study (Fig. 1).

### DAPI band distribution

Numerous DAPI-binding heterochromatic regions were dispersed along all chromosomes and were visible as DAPI dots. These dots, similar to those in chromosomes 5 and 8, did not form distinct DAPI bands. Both the DAPI dots and bands were made more easily visible by inverting the images (Fig. 2c and d).

In addition to the rDNA loci, the presence of several observable DAPI bands along the chromosome complement made identification of homologous pairs possible. The number of the observed bands further increased as the resolution increased after subsequent enhancement of the image sharpness in Genus™. A total of 32 DAPI bands were initially observed in the sharpness-enhanced DAPI images, but six additional DAPI bands were observed after using the inverse tool of Genus™ with adjustment to the brightness and contrast, resulting in a total of 38 bands (Fig. 2b and d).

Twelve of the observed DAPI bands were localized on the short arms, while 26 were on the long arms (Table 2). Among the 24 chromosomes, four had no band (5, 8, 15, and 23), six had one band (2, 3, 9, and 12–14), 11 had two bands (1, 4, 7, 16–22, 24), two had three bands (6 and 10), and one had four bands (11). Furthermore, chromosomes were grouped according to the presence or absence of DAPI bands on each arm (Fig. 3). Group A had no band on either arm (pairs 5, 8, 15, and 23), group B had no band on the short arm, but one band on the long arm (2, 3, 9, and 12), group C had no band on the short arm, but two bands on the long arm (7, 17, and 21), group D had no band on the short arm, but three bands on the long arm (6 and 10), group E had one band on the short arm, but none on the long arm (13 and 14), group F had two bands on both arms (11), and group G had one band on each arm (1, 4, 16, 18–20, 22, and 24).

**Table 2.** Summary of the rDNA and DAPI band distribution patterns.

Chr. no.	rDNA distribution		DAPI band distribution		Remarks
	5S	45S	Short arm	Long arm	
1	-	-	1	1	Pericentric on short arm, more intense intercalary on long arm
2	-	-	-	1	Dispersed, weak,subtelomeric
3	-	-	-	1	Subtelomeric, average intensity
4	-	-	1	1	Pericentric on both arms. Weaker on short arm
5	-	-	-	-	
6	-	-	-	3	One intense pericentric, two intercalary with weaker proximal
7	-	-	-	2	intense pericentric, weak distal
8	-	-	-	-	
9	-	-	-	1	Weak, intercalary
10	-	-	-	3	Weak pericentric, two intercalary with very intense middle and weak distal
11	1	-	2	2	Two moderate intensity flanking 5S rDNA on short arm, one weak intercalary and one weak subtelomeric on long arm. 5S rDNA moderate intensity
12	-	-	-	1	Intercalary, moderate intensity
13	-	-	1	-	Pericentric, weak
14†	-	1	1	-	Subtelomeric on satellite, weak; intense 45S rDNA
15	-	-	-	-	
16	-	-	1	1	Weak subtelomeric on short arm, more intense intercalary on long arm
17	-	-	-	2	Weak pericentric, weak intercalary
18	-	-	1	1	Weak intercalary on short arm, weak pericentric on long arm
19	-	-	1	1	Intercalary on both arms, more intense on short arm
20	-	-	1	1	Intercalary on both arms, more intense on long arm
21	-	-	-	2	Intercalary, proximal more intense than distal
22	-	-	1	1	Intercalary on both arms, more intense on long arm, long arm signal more intense than that on chromosome 20 long arm
23	-	-	-	-	
24	-	-	1	1	Weak subtelomeric on short arm, more intense intercalary on long arm
Total	1	1	12	26	

†satellited chromosome

### Chromosome characterization

In addition to the chromosome length, centromeric position, and rDNA loci distribution, we utilized the observed DAPI bands to characterize the chromosomes. Collectively, these DAPI bands could be very useful in identifying homologues for further cytogenetic analyses, especially of the *Panax ginseng* genome, which comprises a large number of chromosomes with mostly similar sizes. The distinguishing features of each chromosome are presented in Table 2.

## Discussion

There is currently not much genomic or cytogenetic information available for ginseng. Consequently, there are no established cytogenetic markers for the identification of homologous chromosomes. This lack of data has limited our understanding of the karyotype of ginseng and therefore its phylogenetic relationship with other species in the genus *Panax*. In this study, we exploited the usefulness of the 5S and 45S rDNA and the DAPI-binding heterochromatins as molecular cytogenetic markers in pairing homologous chromosomes by analyzing their distribution in the *Panax ginseng* genome.

### Ribosomal DNA and DAPI-binding heterochromatin distribution

We detected only one locus each for 5S and 45S rDNA, which is in agreement with the results reported by Choi et al. (2009). However, the 45S rDNA signal was more intense than the 5S rDNA signal. Owing to the semi-quantitative nature of FISH (Maluszynska and Heslop-Harrison 1991), this could indicate that the 45S rDNA has more repeating units than the 5S rDNA in the ginseng genome.

Localization of the rDNA resulted in our only being able to easily pair two out of the 24 homologues. However, the existence of several DAPI bands distributed along most of the chromosomes greatly facilitated the identification of the other homologous pairs, which otherwise would have been challenging owing to the very low size diffe-rence among most ginseng chromosomes. As a result, DAPI banding, which has been utilized in several previously conducted studies (e.g. Schweizer 1976, Heng and Tsui 1993, Costa Silva et al. 2011),was found to also be an excellent cytogenetic marker in ginseng. Further analysis of the chromosomes based on the DAPI banding pattern on each arm enabled us to categorize them into seven groups (Fig. 3). This technique, which utilizes the presence or absence of DAPI-binding heterochromatin, has the potential for use in future karyotype analyses of ginseng varieties and other *Panax* species.

Chromosomal DAPI bands are caused by the preferential binding of DAPI to AT-rich heterochromatic DNA segments (Schweizer 1976, Eriksson et al. 1993, Heng and Tsui 1993, Kubota et al. 2000) that are long enough to be seen using a fluorescence microscope, suggesting that these DAPI-intense heterochromatic regions in ginseng are AT-rich DNA segments. This information should be useful in the ongoing ginseng genome sequencing because it enables identification of possible characteristics of heterochromatin types present in its genome. Nevertheless, further molecular and cytogenetic analyses are necessary to quantify the AT content of these regions and isolate DNA sequences specific to these heterochromatic bands, like the DAPI-intense signal of the 180-bp knob-specific satellite repeat in maize (Lamb et al. 2007), which is about 56% AT (Peacock et al. 1981, Ananiev et al. 1998).

The use of the rDNA loci number and distribution pattern of other *Panax* species can be useful in deducing the phylogenetic relationship among these species. Choi et al. (2009) showed that wild ginseng and American ginseng (*Panax quinquefolius*), although geographically isolated, have equal numbers of 5S and 45S rDNA loci (2 and 1, respectively), while the cultivated ginseng, although found in the same geographic area with the wild ginseng has only one locus of each rDNA. Although further research is needed to confirm the possible phylogenetic significance of this report, we found only one locus for each type of rDNA in all three cultivars and one local landrace of *Panax ginseng*.

### Ginseng karyotype and ploidy

Karyotype data are essential to understanding the phylogenetic relationships among species belonging to the same family (Heslop-Harrison and Schwarzacher 2011, Mendes et al. 2011), making them useful to cyto-taxonomic studies (Pinto et al. 2012). Additionally, comparative cytogenetics provide knowledge regarding the cytogenetic relationships between diploid species and their polyploid cytotypes, as well as between allopolyploids and their ancestral genomes (Kovarik et al. 2005, Leflon et al. 2006, Snowdon 2007, Wang et al. 2007, Kolano et al. 2008, Xiong and Pires 2011).

Most species belonging to the family Araliaceae are 2*n*=24 or 2*n*=48, except for a few genera that have little chromosomal number variation (Yi et al. 2004). In a review of the chromosomal evolution of the family Araliaceae, Yi et al. (2004) discussed that, although the actual basic chromosome number of the family was thought to be *x*=12, some species were 2*n*=36. These species would be triploids if the basic chromosome number 12 is considered, but triploids are genetically unstable. This caused a challenge in establishing the basic chromosome number of the family. The *x*=12 hypothesis was further challenged after the genus *Hydrocotyle* which has several taxa with 2*n*=18, 36, and 60 were moved into Araliaceae form Apiaceae, giving an alternative basic chromosome number *x*=9 and *x*=6. Nevertheless, *x*=12 is generally accepted as the basic chromosome number in the family, but this does not eliminate the possible ancestral *x*=6 (Yi et al. 2004). One hypothesis cannot easily rule out the other but further phylogenetic and karyotype analyses in the family are necessary to resolve these competing hypotheses.

Considering a basic chromosome number of 12 or 6, ginseng would be considered a tetraploid or octoploid, respectively; the latter having a more ancient nature. Recently, Choi et al. (2011) showed the high replication of homologous genes in ginseng using SSR markers and suggested that the polyploidy could range from tetra- to octoploidy. Nevertheless, in practice, *Panax ginseng* is regarded as a tetraploid species with a basic chromosome number of 12 (Wen and Zimmer 1996, Court 2000, Yi et al. 2004, Choi et al. 2009).

Our data showed a somatic cell chromosome complement of 2*n*=48, supporting previously reported chromosome numbers (Ko et al. 1993, Choi et al. 2009) and polyploidy (Wen and Zimmer 1996, Court 2000, Yi et al. 2004, Choi et al. 2009). However, evaluation of the rDNA loci number revealed only one locus for each 5S and 45S rDNA, despite its polyploid nature. This reduction of rDNA loci may be explained by the non-additive nature of rDNA loci and other genomic DNA segments after polyploidization (Snowdon et al. 1997, Ozkan et al. 2003, Yoshikazu et al. 2006). More over, loss of the duplicate loci may be brought about by single-generation or rapid genome/chromosomal reorganization (Wendel 2000, Heslop-Harrison and Schwarzacher 2011), or from the gradual action of concerted evolution after genome duplication or alloploidization (Kovarik et al. 2005). In the former case, it would be difficult to tell whether ginseng is an ancient polyploid, while in the latter, the loss of these loci would provide obvious evidence of an ancient polyploidization event. However, some species do not really reflect a correlation between the rDNA loci number and the level of ploidy; in fact, polyploids can even have half the number of rDNA signals than their diploid counterparts (Yoshikazu et al. 2006). This rDNA reduction phenomenon has been well-documented in the *Artemisia* species (Pellicer et al. 2010).

Additionally, based on localization of the 45S rDNA near the centromere area and the intercalary position of the 5S rDNA, it is just as likely that these loci were favored to survive locus loss from non-additive recombination over their duplicated counterparts, which probably would have been in more distal positions, or epigenetically silenced (Kovarik et al. 2008).

## Conclusion

The first report of *Panax ginseng* karyotype using ribosomal DNA and DAPI bands as cytogenetic markers is presented here. The presence of long stretches of DAPI-binding heterochromatin was useful in the detailed karyotyping. The results presented here will be useful in further cytogenetic analyses and the on-going genome sequencing of ginseng. More cytogenetic research is needed to understand the cytogenetic history of ginseng and other species in the genus *Panax*. Further comparative cytogenetic analyses among its close relatives will provide more insight, and further genomic analyses of the heterochromatin distribution will enhance our knowledge of its genomic history.
